# Bisphenol A release from food and beverage containers – A review

**DOI:** 10.1002/fsn3.3398

**Published:** 2023-05-07

**Authors:** Elham Khalili Sadrabad, Sayed Aliasghar Hashemi, Azadeh Nadjarzadeh, Elaheh Askari, Fateme Akrami Mohajeri, Fereshteh Ramroudi

**Affiliations:** ^1^ Research Center for Food Hygiene and Safety, Department of Food Hygiene and Safety, School of Public Health Shahid Sadoughi University of Medical Sciences Yazd Iran; ^2^ Shahrekord University Shahrekord Iran; ^3^ Nutrition and Food Security Research Center, Department of Nutrition, School of Public Health Shahid Sadoughi University of Medical Sciences Yazd Iran; ^4^ Nutritional Health Research Center, School of Health and Nutrition Lorestan University of Medical Sciences Khorramabad Iran; ^5^ Infectious Diseases Research Center, Shahid Sadoughi Hospital Shahid Sadoughi University of Medical Sciences Yazd Iran

**Keywords:** bisphenol A (BPA), BPA migration, BPA release conditions, foodstuff

## Abstract

Dietary exposure was introduced as the primary way Bisphenol A (BPA) enters the human body. Although significant efforts have been made to analyze BPA's presence in different foodstuffs, less attention has been given to introducing the conditions that facilitate BPA release. This review aimed to mention possible factors affecting BPA release into foods and beverages. According to the results, the critical factors in BPA release are temperature, manufacturing process, food and packaging type, pH, mineral elements, repeated use, irradiation, washing, contact time, and using detergents. It showed that using PC containers, high temperature and pH, storage under solar irradiation, alkaline detergents, water hardness, and repeated use could increase the BPA release from containers into foodstuff. During various conditions, hydrolysis of the carbonate linkage and *d*‐spacing will increase. Considering these parameters and limiting the use of PC containers, the potential risk of BPA exposure could be eliminated.

## Graphical abstract

Repeated use, washing, and rinsing methods with boiling water, type of detergents, alkaline condition, type of food and packaging, manufacturing process, and irradiation could be effective in BPA leaching. It is recommended to substitute the PC with safer packaging, avoid storage of bottled water under solar irradiation and outdoors, and using boiled water in baby bottles. The manufacturer should state the cleaning and sterilization methods and discarding condition of products on the label of bottles or packaging.

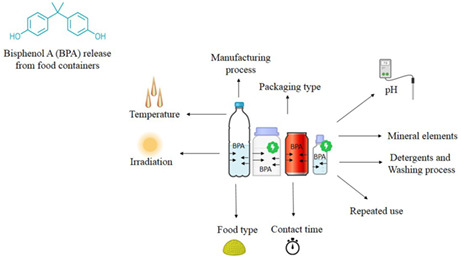

## 1 INTRODUCTION

Bisphenol A, known as 2,2‐(4,4‐dihydroxy diphenyl) propane, is an organic compound with two phenol rings and two methyl functional groups (Kang et al., 2006). Bisphenol A (BPA) is a water‐soluble compound from a group of diphenylmethane derivatives (Vilarinho et al., 2019). The BPA produces six billion pounds each year (Vandenberg et al., 2007), which is expected to increase by 6%–10% each year (Maia et al., 2009). The BPA is widely used as a constituent of polycarbonate plastics and epoxy resins and as an antioxidant and inhibitor of polymerization in polyvinyl chloride plastics (PVC; Geens et al., 2012; Maiolini et al., 2014; Nam et al., 2010). According to research, the primary way of bisphenol exposure for people is through food and beverages contaminated with BPA (Maiolini et al., 2014). Several studies indicated that BPA could be released from polycarbonate containers into food and water under normal use conditions (Li et al., 2010; Maia et al., 2009). The BPA migration process consists of physical and chemical migration. Physical migration is due to the diffusion of residual BPA monomers after manufacturing. In contrast, chemical migration is a consequence of BPA release from the polymer surface, mainly caused by hydrolysis (Hao, 2020). Therefore, the presence of BPA in foodstuffs could be attributed to the migration of residual BPA or hydrolysis of the polycarbonate (Nam et al., 2010). It should be mentioned that the term “migration” in food packaging refers to the whole process of the release or transfer of compounds from packaging into the food (Mercea, 2009).

The BPA imitates the estrogenic hormones (17 β‐estradiol) even at low doses, which could increase hormonally mediated cancers (Maia et al., 2009, 2010) and disrupt endocrine activity (Le et al., 2008). The estrogenic capacity of bisphenols is related to hydroxyl groups in the para position (Vilarinho et al., 2019). Also, some adverse effects such as metabolic disorders, obesity, autism, decrease in antioxidant enzymes, endometriosis, cardiovascular disease, reproductive cancers, fertility problems, neonatal mortality, multiple ovarian cysts, sexual dysfunction, and attention deficit hyperactivity disorders have been reported (Baluka & Rumbeiha, 2016; Errico et al., 2014; Maia et al., 2010; Vandenberg et al., 2007). It was shown that the fetus is sensitive to the adverse effects of BPA due to passage from the placenta and a lower concentration of UDP‐glucuronosyltransferase in the fetus (approximately five times lower than in adults; Kang et al., 2006). The presence of BPA in the environment and the human body has convinced scientists to assess the potential health risk associated with BPA (De Coensel et al., 2009). The European Food Safety Authority (ESFA) and FDA set a daily intake of 4 μg BPA/Kg/day and 50 μg BPA/Kg per day, respectively (Hao, 2020). The BPA limit was set as 600 μg/kg by the EU Commission (Errico et al., 2014). The adverse effects of BPA have been reported even at very low doses (Borrirukwisitsak et al., 2012). This review aimed to summarize the different parameters accelerating BPA release in foodstuffs.

## 2 MATERIALS AND METHODS

The eligible studies investigated the effect of different procedures in BPA migration or release between English publications from 2005 to 2023 (February) and were included in the current review research. The search was done in different database such as PubMed, Sciences Direct, Web of Science, Google scholar, and Scopus using key words “bisphenol A (BPA)” and “alkylphenols,” “residual BPA,” “monomer residues” companion with other terms such as “migration,” “release,” “leaching,” “brands of bottles,” “baby bottles,” “bottled water,” “polycarbonate bottles (PC),” “polyethylene terephthalate (PET) bottles,” “Tritan™ bottles,” “non‐PC bottles,” “free BPA bottles,” “reusable bottles,” “stainless steel water bottles,” “aluminum water bottles,” “barreled drinking water,” “plastic cup,” “salinity,” “mineral elements,” “pH,” “alkalinity,” “irradiation,” “sun light,” “temperature,” “heating,” “microwave heating,” “boiling,” “steaming,” “cooking,” “sterilization,” “thermal degradation,” “contact time,” “incubation time,” “packaged food,” “canned foods,” “canned fruits,” “canned vegetables,” “canned seafood,” “canned beverage,” “canned drink,” “milk,” “infant formula,” “amines,” “ food simulant,” “fatty acid simulant,” “dishwashing,” “storage condition,” “real use,” “severe conditions,” “repeated use or aging,” “photoaging,” “manual washing,” “brushing,” “detergent.” The inclusion criteria for this study were all studies whose full text is available. They investigated the effect of different conditions in the release and migration of BPA from containers into food and beverage. Exclusion criteria also included all studies that the full text is not available, and analyses presented as abstracts in scientific conferences are excluded from the main study. The titles and abstracts of articles extracted from databases are reviewed in the review process, and final studies are classified according to their relevance to the manuscript subject. Two researchers extracted the data according to standard form, and the differences were discussed by a third party. The article's information, including publication year, first author name, type of containers, type of examined procedures for containers, level of BPA released, and critical findings, were extracted.

## 3 BPA RELEASE CONDITIONS

The different studies worked on different conditions leading to BPA release. There are different conditions such as heating and boiling, brushing, container manufacturing process, packaging type, exposure to food acidity or alkalinity (pH), aging of the container, food storage temperature, irradiation, salt, glucose, oil, and using detergent which could facilitate the BPA release and migration into foodstuff (Ali et al., 2019; Osman et al., 2018; Shrinithivihahshini et al., 2014). Based on different studies, essential parameters of BPA release are categorized as follows. On the other side of the spectrum, due to overlapping factors, their categorizing seems complicated.

### 3.1 Manufacturing process

Research shows that trademarks and packaging should be considered a significant factor in BPA migration from PC polymers (Errico et al., 2014; Santillana et al., 2013). Different conditions in commercial bottle manufacturers, such as molding condition, annealing process, residual BPA in the resin, moisture content, and installation to draw water and to fill the bottles, could lead to BPA release into the container content (Ginter‐Kramarczyk et al., 2022; Mercea, 2009). In the manufacturing process, the airborne BPA could be blown and entered into plastics during the air expansion process (Holmes et al., 2021). Therefore, the presence of BPA could be attributed to residual BPA on the bottle surface (Maragou et al., 2008). The process of manufacturing (incomplete polymerization) and technology of bottling (migration from materials in tanks and pipes), cross‐contamination, as well as the quality of raw material could result in the scattered presence of BPA (Amiridou & Voutsa, 2011; Guart et al., 2014; Kumar et al., 2023). The results of Agarwal et al. (2022) indicated that local PC cups had higher BPA release than branded ones which was attributed to a higher amount of nonpolymerized compounds and lower hydrothermal stability of locally produced cups. Errico et al. (2014) illustrated that the high leaching of BPA in cherry tomatoes compared to other tomato products is due to different processing conditions, and the thickness of the metal can change the coating. Fan et al. (2014) showed that all 16 brands of PET bottles release BPA in the range of 37.7–64.1 μg/L. The study in Cameron revealed that one brand of baby bottle out of 14 brands had BPA release in the concentration of 61.2 μg/L after the first washing (Olivier et al., 2023).

### 3.2 Packaging type

Food packaging is an excellent way to protect food from the outer environment and preserve its nutritional value (Fabusola & Akpambang, 2021), although chemical compounds, including BPA, could leach from food packaging into food (Elobeid et al., 2012). Noting the studies' findings, polymers used in products are necessary for the BPA release (Mansilha et al., 2013). Different packaging materials used in food and beverage include polycarbonate, polyethylene terephthalate, high‐ and low‐density polyethylene, metallic cans, polystyrene, polypropylene, and polyvinyl chloride.

#### 3.2.1 Polycarbonate packaging

Polycarbonate with high transparency, high heat resistance (−40 to 145°C), and low weight are widely used in the food industry as reusable containers for water bottles, baby bottles, kitchen appliances, canned foods, microwave ovenware, plates, storage containers, and beverages (Geens et al., 2012; Maia et al., 2009). The polymerization of BPA with diphenyl carbonate or carbonyl chloride resulted in the production of PC (Esmer et al., 2017). The presence of BPA in PC samples was explained by two different procedures of polymer aminolysis/hydrolysis and BPA residual diffusion (unreacted residual BPA), which could be enhanced in different conditions (Esmer et al., 2017; Geens et al., 2012). The European Commission (EC) set the law to forbid the use of PCs in baby bottles (Commission E, 2011). According to Onn Wong et al. (2005) findings, BPA was detected in 19 PC baby bottles with a mean concentration of 28.1 mg/kg. Cooper et al. (2011) research evaluated the BPA migration from PC bottles at 0.2–0.3 mg/L at room temperature. In the study by Ali et al. (2019), some PC baby bottles with free BPA labels showed BPA release under brushing procedures. In Ehlert et al. (2008) research, the BPA residue ranged from 1.4 to 35.3 mg/kg. The variation in surface characteristics of PC samples led to different concentrations of PC chain ends and variations in the BPA migration (Mercea, 2009).

#### 3.2.2 Polyethylene terephthalate packaging

Polyethylene terephthalate (PET) is a usual polymer used in the packaging of bottled drinking water (Bach et al., 2013). PET comprises ethylene glycol and terephthalate acid without BPA monomers (Yun et al., 2008). Although PET containers had limited additives and a lower diffusion rate of harmful compounds, background contamination, water source, prebottling, and recycling process of PET could contaminate the containers with BPA (Bach et al., 2013; Cao & Popovic, 2018; Fan et al., 2014; Hao, 2020). Yun et al. (2008) indicated that the BPA concentration of PET bottled water was evaluated as higher than PC ones. It was assumed that the caps of PET bottles were a potential BPA source. On the contrary, BPA was not detected in the PET samples of Guart et al. (2011) research. In another study by Guart et al., BPA was detected in PC, PET, and glass bottles with metallic crown caps (Guart et al., 2014). In the research by Wang et al. (2020), the BPA concentration in PET bottled waters was measured 67 times lower than in PC ones, which was attributed to PC packaging material and reuse times. The migration of BPA from food‐grade PET could be attributed to the recycling process of PET, which increased the BPA (Hao, 2020). In general, it is worth noting that BPA release in packaging such as PET, glass, and HDPE is lower compared to PC packaging (Osman et al., 2018).

#### 3.2.3 High‐density polyethylene and low‐density polyethylene

High‐density polyethylene (HDPE) is a resistant container with a volume of 5–8 L, which could also be used as a cap material (Guart et al., 2011). In Guart et al. (2011) study, BPA was not detected in HDPE bottles, while BPA in the concentration of 0.145 μg/dm^2^ was evaluated in HDPE caps. Low‐density polyethylene (LDPE) has the same structure as HDPE, with lower density used in caps. The BPA migration from LDPE was evaluated as 0.128 μg/dm^2^ (Guart et al., 2011). According to Guart et al. (2014) results, BPA was detected in 60% of fresh waters and 95% of 1‐year storage waters stored in PC bottles with LDPE cap.

#### 3.2.4 Metallic cans

The inner surface of metallic cans is coated with a thin layer of epoxy resins or organosols. Accordingly, uncompleted polymerization of epoxy resins and food canning process could lead to leaching and migration of BPA into food (Errico et al., 2014). During the canning process, sterilization of canned foods could lead to the diffusion of BPA (Osman et al., 2018). Also, the nature and type of inner coating and geometry influence the BPA migration (Noureddine El Moussawi et al., 2018). It is worth noting that the type of canned foods is essential in BPA migration. The powdered infant formula cans, which had no coatings or layers, showed low BPA release compared to liquid infant formula cans (Cao et al., 2008). Lorber et al. (2015) determined that BPA in canned food samples was much higher than in noncanned foods. However, Kumar et al. (2023) showed that the low concentration of BPA in canned soft drinks ranged from 8.91 to 14.01 pg/mL. Kovačič et al. (2020) corroborated that beverage cans and reusable steel bottles, owing to organic coating and the linings, had the highest concentration of BPA. In line with other researchers, Liao and Kannan asserted that canned foods, especially fish and seafood, fruits, beverages, and condiments, had the highest BPA concentration; its concentration in canned foods is two to four times higher than plastic and paper containers (Liao & Kannan, 2014). The research on eight food categories revealed that foods packaged in cans or plastic bags showed higher BPA concentrations attributed to the leaching of packaging materials (Adeyi & Babalola, 2019). According to the results of Geens et al. (2010), the BPA concentration in canned foods was 40 times higher than in canned beverages. Generally, the variation in BPA of canned foods is related to can coating material and thickness, temperature and duration of can sterilization, storage and transportation condition, country of origin, manufacturer, and analysis method (Cao et al., 2009; Cunha & Fernandes, 2013; Fan et al., 2014; Geens et al., 2010). Also, the lower BPA levels in canned fruits compared to canned vegetables were attributed to applying electrolytic tinplate in fruit containers instead of epoxy films (Cunha & Fernandes, 2013). Grumetto et al. (2008) reported insignificant differences in the bisphenol content of cans coated by epoxy phenolic or low BADGE. Braunrath et al. (2005) displayed that except for energy drinks, all studied canned foods contain BPA in concentrations lower than the EU limit.

#### 3.2.5 BPA‐free packaging

In recent years, the application of substituted PC plastic bottles called “BPA‐free” has been studied. The lined aluminum water bottles are introduced as reusable and safe water bottles. However, polymerization and chemical reactions of BPA derivatives used in aluminum bottle linings resulted in the release of unmodified free BPA (Cooper et al., 2011). Also, Tritan™, a copolyester polymer, is introduced as a BPA‐free water bottle. The research on the effect of washing on the release of BPA from Tritan™ plastics indicated that the presence of BPA is likely due to surface contamination of the manufacturing process, which could eliminate the BPA release (Holmes et al., 2021). A study by Cooper et al. (2011) revealed that BPA was not detected in Tritan™ and uncoated stainless steel water bottles. Simoneau et al. (2012) indicated that bottles made of polyethersulfone and Tritan™ had no release of chemical substances. Otherwise, polypropylene and silicone bottles showed higher chemical substance migration. The BPA was detected in baby bottles made of polyamide.

#### 3.2.6 Other types of packaging

Polystyrene (PS) plastics used in caps are hard and transparent products. According to Guart et al. (2011) study, BPA was detected at 0.136 μg/dm^2^ in PS samples. In Kubwabo et al. (2009) study, the non‐PC bottles (polysulfone, polypropylene, and polystyrene) had lower BPA release compared to PC ones. Indeed, the presence of BPA in non‐PC bottles was associated with the manufacturing process. According to the result of Hananeh et al. (2021), plastic cups with SPI code 5, made of polypropylene, could release BPA in contact with hot beverages. The BPA level was below the standard limit in all glass bottles, vat, and tetra Brik wine samples. It was shown that vat material, storage time, type of wine (red or white), alcohol concentration, packaging material, and top of the glass bottles had no significant influence on the BPA concentration (Brenn‐Struckhofova & Cichna‐Markl, 2006). In Hao's research, BPA was found in 60% of barreled drinking water, attributed to raw water and noneffective removal of BPA during processing, migration, and release from the barrel (Hao, 2020). In Kumar et al. (2023) study, the highest BPA concentration in plastic and concrete water tanks was estimated at 12 pg/mL. It was shown that BPA is used in food packaging PVC films to remove surplus hydrochloric acid. Therefore, the presence of BPA in cheese samples in Cao et al. (2011) research was attributed to the probable migration of BPA from plastic packaging films and cross‐contamination during processing. In the study by Astolfi et al. (2021), the BPA in stainless steel, aluminum, recycled, plastics, and silicone water bottles was evaluated as lower than the limit of the detection.

### 3.3 Food type

According to investigations, the baby bottles containing infant formula showed greater BPA release than food simulants (50% ethanol and 3% acetic acid; Santillana et al., 2013). Due to the solubility of BPA in ethanol, migration in the water–ethanol mixture could be increased, which was attributed to the back migration of BPA resulting from the hydrolysis (Mercea, 2009). The BPA presence in baby foods in the jar in Cao et al. (2011) research was attributed to cross‐contamination during processing by epoxy coating equipment, PVC gaskets, and epoxy coating of metal lids. Johnson et al. (2015) reported that baby bottles containing milk samples had slight BPA leaching compared to water and apple juice, which was attributed to the solubility of BPA in milk fatty materials. Biedermann‐Brem and Grob (2009) indicated that the BPA release in milk‐type beverages was lower than in tea due to the neutral pH made by protein. According to Cao et al., most dairy samples had not contained BPA. The presence of BPA in canned evaporated milk (15.3 ng/g) was attributed to the can coating migration (Cao et al., 2011). On the other hand, Osman et al. (2018) reported that the high amine content of milk (putrescine and 1,4‐diamino butane) could simulate the BPA migration. It was mentioned that compounds with low molecular weight and amine groups presented in milk or infant formula could catalyze the depolymerization process (Santillana et al., 2013). Therefore, the degradation of polycarbonates could lead to BPA migration in the environment (Benhamada et al., 2016). The BPA in 21 samples out of 30 canned beverages and two pieces out of seven powdered infant formula was detected in Portugal (Cunha et al., 2011). The level of BPA varied by alcohol content, manipulation during food processing, and species composition of the food matrix; for example, lipid content showed higher levels (Cabado et al., 2008; Fasano et al., 2015). However, having investigated that nonaqueous nonpolar liquid such as olive oil cannot hydrolysis the PC and solve BPA (Mercea, 2009). Apart from food simulants, the nature of water (pH and mineral content) is considered necessary in the BPA migration (Mercea, 2009). Carbonated waters, due to the production of carbon dioxide, could decrease the pH of the water to 6, which improves the plastic degradation and migration of BPA into the waters (Guart et al., 2014). Although the carbonated waters are often packed in glass containers, the metallic crown caps are responsible for the presence of plasticizers and additives (Guart et al., 2014). It has been mentioned that apart from BPA leaching from packaging and foods, human activities on aquatic life lead to environmental pollution in the aquatic environment and mineral water (Adeyi & Babalola, 2019; Bingol et al., 2018).

By comparison of solid and aqueous fractions of foods, it was revealed that BPA is more distributed in the solid fractions. Indeed, there is an assumption that the presence of BPA in the fat content of solid fractions is higher than in aqueous foods (Geens et al., 2010). It was suggested that oils in tuna canned foods could facilitate BPA migration from the can lining (Fattore et al., 2015). Cao et al. (2011) detected low BPA levels in most fast food samples. The BPA in hamburger samples was measured at 10.9 ng/g for wrapping paper and its ingredients, including cheese, sauce, beef, and bread. The BPA migration in food containing a high amount of protein and fatty foods such as meat, fish, and coconut cream has been reported (Osman et al., 2018). However, in Cao et al. (2011) study, marine, freshwater fish, and shellfish samples contained a low amount of BPA. According to Adeyi and Babalola's findings, the BPA concentrations were in the order of aquatic foods > edible oils > meat products > dairy products > vegetables > fruits > chicken eggs = cereals. It was explained that foods in plastics and can packaging had the highest BPA concentration. In the foods, including raw beef, chicken, cheese, apple, tomatoes, beans, rice, and chicken eggs, the BPA was not detected (Adeyi & Babalola, 2019). Fasano et al. (2015) indicated that the lowest BPA was estimated in broth and wines, which were attributed to the composition of packaging. It was mentioned that BPA migration was considered higher in fatty food simulants than in aqueous food after the heat process (Munguia‐Lopez et al., 2005). Some reports show that sodium chloride solutions could increase the leaching of the BPA (Vandenberg et al., 2007). The low level of BPA in cereal and bread products was due to the presence of BPA in baking powder and yeasts (Cao et al., 2011). The highest BPA migration in Onn Wong et al. (2005) was reported in 10% ethanol at 70°C (0.64 μg/in^2^) and corn oil at 100°C (0.43 μg/in^2^).

### 3.4 pH

It was shown that with the presence of hydroxyl groups in BPA diglycidyl ether, their stability could be affected by the pH (Bingol et al., 2018). Regarding studies, the PC hydrolysis could be accelerated by increasing the pH (Nam et al., 2010). In other words, the pH could affect the packaging stability (Bingol et al., 2018). It was confirmed that degassing carbon dioxide from tap water by boiling increases the pH and release of the BPA (Biedermann‐Brem & Grob, 2009). Biedermann‐Brem et al. (2008) showed that using 3% citric acid lowered the BPA formation by 10 times. Also, lime in a lack of bicarbonate solution leads to BPA liberation (Biedermann‐Brem & Grob, 2009). Benhamada et al. (2016) showed the BPA concentration of 78, 137, and 311 μg/L in acid, neutral, and essential solutions, respectively. So, it was confirmed that by increasing pH, the release of BPA will increase (Benhamada et al., 2016; Mercea, 2009).

### 3.5 Mineral elements

The findings illustrated that water hardness could increase PC packaging degradation and BPA release (Bingol et al., 2018). In the presence of salts, including sodium bicarbonates, carbon dioxide releases lead to pH rise and BPA formation (Biedermann‐Brem et al., 2008). Vilarinho et al. (2019) confirmed that hard water could increase the BPA release by raising the pH. Mercea studied the effect of adding neutral salts (Na^+^, Ca^2+^, and Mg^2+^) in PC bottles (Mercea, 2009). It was shown that the BPA migration was elevated by increasing the temperature and mineral content. Also, this increase in tap water was slower since the carbon dioxide will evaporate during boiling, and changes in the equilibrium toward carbonate occur. Its precipitation leads to a delay in pH increase (Biedermann‐Brem & Grob, 2009). Also, the BPA migration was increased in the presence of Na^+^ and high pH values. Taking the temperature and pH constant made these interactions small, and PC hydrolysis was not catalyzed by cations (Mercea, 2009). Therefore, changing the physic‐chemical properties of water (pH, conductivity, Ca, and Mg content) could increase or decrease the risk of BPA contamination (Bingol et al., 2018). It should be pointed out that the application of ion exchangers in household installations for water decalcification resulted in a rapid pH increase. This decalcification remains carbonate or bicarbonate in water, decreasing the carbonate participation rate (Biedermann‐Brem & Grob, 2009).

### 3.6 Repeated use or aging

Different studies showed that aging and repeated use increase the wettability of the container's wall (Ali et al., 2019; Benhamada et al., 2016; Santillana et al., 2013), weaken the polymer surface (Shrinithivihahshini et al., 2014), increase the *d*‐spacing of the PC (Benhamada et al., 2016), and increase the BPA migration. In other words, the different treatments on the PC lead to decreasing the molecular weights by breaking the chains (Benhamada et al., 2016). Also, it was indicated that the remaining high amount of liquids on the bottle surface, after washing, would increase wettability and liquid evaporations, leading to more carbonates (Biedermann‐Brem et al., 2008). In other words, erosion of the bottle surface during repeated use could accelerate the BPA migration due to the expansion of the contact area (Cao et al., 2013). The presence of BPA is not only due to the diffusion mechanism of nonpolymerized monomers but originated from the degradation of PC (Benhamada et al., 2016). In recent years, using amines and amides as a possible way to recycle plastics through aminolysis of polycarbonate has been used (Maia et al., 2010). Moghadam et al. (2015) reported the BPA to release with the concentration of 0.49–8.58 and 0.63–2.47 μg/L for new and used baby bottles, respectively. Nam et al. (2010) showed that BPA migration in PC baby bottles reached 3.08 ppb after repeated 100‐time uses (the equivalent to 5 months of use) due to expanding the average distance of PC chains. It was reported that EU repetitive‐use conditions overestimated approximately 10 times higher than the normal daily use of baby bottles (Onghena et al., 2016). As can be seen from studies, regrinding and remolding used PC containers would cause an increase in the BPA migration (Mercea, 2009).

### 3.7 Irradiation

It was indicated that ultraviolet light and oxidizing in the air could accelerate the aging process and BPA release from PC baby bottles (Nam et al., 2010). Storage of bottled waters outdoors and under solar irradiation results in the photolytic formation and degradation of organic compounds via direct or indirect photoreactions (Amiridou & Voutsa, 2011; Elobeid et al., 2012). Results of Esmer and Çağindi (2020) showed that storage of PC water bottles under sunlight at 20°C increased the BPA migration. In Yun et al. (2008) research, the BPA concentration in PET bottled water reached 45.1 ng/L after 8 days of storage in the car under sunlight. In Parto et al. (2022) research, the BPA was increased with storage time and exposure to sunlight. Ugboka et al. (2022) reported an increase in the BPA release of bottled water and carbonated drinks after 60 days of storage under sunlight. Therefore, it seems essential to protect drinking water bottled in cool places and far from solar radiation (Elobeid et al., 2012). Park et al. reported that by increasing the doses of gamma‐irradiation to 30 kGy, the BPA release in PC films was raised and remained constant at 60, 100, and 200 kGy. It was explained that the cross‐linking effect would be predominant at 5–10 kGy doses of gamma‐irradiation. By increasing the irradiation from 10 to 30–200 kGy, the cleavage in weak carbonyl bonds in phenyl rings would happen. The chain scission and cross‐linking caused the BPA formation from the polymers (Park et al., 2006). On the other hand, microwave heating was not the necessary procedure for BPA release. Onghena et al. (2016) indicated that 100 cycles of microwave heating released a low concentration of BPA. It was shown that microwave heating (800 W for 3 min) had insignificant effects on BPA migration. Factors such as the initial food contact with plastic containers (polypropylene and polycarbonate plastic) and the cooking process are introduced for BPA detection in samples. It was explained that the microwave's ability to water heat the core of the food instead of the food contact surface is responsible for lower contaminant migration (Fasano et al., 2015). Ehlert et al. (2008) also confirmed that three microwave heating cycles did not affect BPA migration from PC baby bottles into water. However, comparing boiling water in a pan and microwave revealed that BPA liberation was higher in microwave heating, since microwave heating degassing and carbon dioxide removal are more intense (Biedermann‐Brem & Grob, 2009). Lim et al. (2009) showed that heating fat food simulants, steamed hot rice, and cooked hot pork by microwave resulted in BPA migration from PC containers. The variation in BPA release during microwave heating could differ by test condition and contact duration (Agarwal et al., 2022).

### 3.8 Temperature

According to studies, in high temperatures, the carbonate linkages are affected by the hydrolytic attack of the ester bands, and the d‐spacing of PC containers increases (Ali et al., 2019; Fasano et al., 2012; Nam et al., 2010). A positive relation was reported among BPA migration, temperature, and the number of baby bottles containing milk uses (Osman et al., 2018). Maragou et al. indicated that migration of BPA was only detected in bottles filled with boiling water at the 16th cycle of brushing and sterilization. According to their result, temperature was introduced as a crucial factor in BPA migration. These researchers showed that aqueous depolymerization occurs in severe conditions (Maragou et al., 2008). It was reported that sterilization of baby bottles with boiling water mainly increased the BPA release (Esmer et al., 2017). Therefore, using hot water in PC baby bottles is enough to hydrolyze and release BPA (Nam et al., 2010). In Nam et al. (2010) research, by increasing the temperature from 40 to 95°C, the BPA migration increased four times in new baby bottles. Benhamada et al. (2016) reported undetectable BPA at 20°C, medium at 50°C, and high at 100°C. Biedermann‐Brem and Grob (2009) reported that baby bottles filled with boiling water had three μg/L BPA with an increased pH value of 8.3. The research on plastic cups made of polypropylene containing hot Arabian coffee indicated migration of BPA during a short period (Hananeh et al., 2021). Lim et al. showed that storing steamed hot rice or fat food simulant (meat) in PC containers and heating in a boiling bath for 30 min had no BPA migration. However, the low levels of BPA were detected by microwave heating in steamed hot rice or fat food simulant (meat; Lim et al., 2009). Lim et al. (2009) confirmed that heating by microwave or storing boiling water could increase the BPA migration. Onghena et al. (2016) showed that the BPA migration was reduced after microwave, steam, and cooking sterilization and dishwashing. According to their results, steam sterilization was recommended for daily use due to the lower release. El Moussawi et al. (2019) showed that despite pasteurization, the sterilization of canned food resulted in BPA release in the food, although in Maragou et al. (2008) research, sterilization by boiling water for 10 min did not affect degradation. The presence of BPA in canned food was attributed to sterilizing process and storage factors (time and temperature; Osman et al., 2018). These variations in achieved results are due to temperature and contact duration. The high temperature with an increased contact period increases the BPA migration due to the hydrolysis of PC polymers and release of nonreactive monomers (Hananeh et al., 2021; Kubwabo et al., 2009; Mansilha et al., 2013). It can be observed that high temperature, prolonged contact time, pressure, and alkali medium could increase depolymerization (Benhamada et al., 2016; Maragou et al., 2008).

In canned food especially low‐acid canned foods, commercial sterilization is introduced as an essential factor in the BPA release (Munguia‐Lopez et al., 2005). According to Fabusola and Akpambang (2021), the BPA in canned tomatoes ranged from 0.124 to 0.141 mg/kg. It was shown that BPA concentration in heated cans (100°C) was approximately 1.7–55.4 times higher than in unheated ones (Vandenberg et al., 2007). Therefore, heating could facilitate BPA release from cans (Noureddine El Moussawi et al., 2018). In line with the effect of processing conditions, Cao et al. (2009) indicated that the BPA concentration in 85% of canned soft drinks was below 1 μg/L, which confirms the slow BPA release of can coating at room temperature storage. However, it will be assumed that the presence of BPA in raw, canned vegetables could also be related to the canning process (El Moussawi et al., 2019).

### 3.9 Contact time

The migration of BPA into food is proportional to the square root of contact time. It was shown that contact period and contact temperature could increase the BPA migration (Esmer & Çağindi, 2020; Kubwabo et al., 2009; Lim et al., 2009; Onn Wong et al., 2005). Fan et al. (2014) indicated that the release rate of BPA decreased with storage time which may become stable under long‐term storage. According to Guart's results, BPA in first incubation period was due to the manufacturing process, and no BPA was detected in the second and third incubation periods (Guart et al., 2013). Therefore, after warming infant formula in PC bottles, the contact time should be deliberated to keep a minimum (Cao & Corriveau, 2008). Fan et al. indicated that the BPA concentration of PET bottles was increased during storage at 70°C for 4 weeks. However, it was shown that BPA concentration remains stable during long storage contact time (Fan et al., 2014). In Dehdashti et al. (2023) research, the increase in BPA was reported during initial storage, which decreased over time. Geens et al. (2010) showed that BPA in the liquid fraction of food decreased during incubation time while increasing in the solid phase. Benhamada et al. (2016) showed that as the treatment duration increased, the BPA concentration in PC samples increased. The decrease in the BPA concentration of canned food during storage was attributed to inter‐cans variability (El Moussawi et al., 2019). On the other hand, the BPA reduction after 10 days of storage at 60°C was attributed to BPA repartitioning in the coating (Noureddine El Moussawi et al., 2018). Also, Munguia‐Lopez explained that the gradual decrease of BPA in heated cans could be attributed to the activity of BPA as an antioxidant in preventing oil oxidation (reaction with free radicals) initiated by the heat process (Munguia‐Lopez et al., 2005).

### 3.10 Using detergents and the washing process

The washing of baby bottles is an ordinary procedure which could be accompanied by detergent and hot water (Maia et al., 2009). Maia et al. investigated the release of BPA in baby bottles immersed in different detergents and bleach. The result indicated that the BPA does not result from diffusion and is considered the primary product of interaction between detergent and PC at high temperatures. Therefore, it was shown that using detergent could increase the BPA concentration even after rinsing (Maia et al., 2009). Ali et al. (2019) indicated that by 60 cycles of brushing, the BPA release was increased in all studied baby bottles. On the other hand, Mansilha et al. (2013) reported that BPA concentration in manually cleaned water was significantly lower than in washing with a dishwasher. This could be explained by the fact that detergents mainly used in washing machines are alkali compared to manual washing (Biedermann‐Brem et al., 2008). Also, by the increase in the detergent concentration in the drying process, the BPA release was improved (Biedermann‐Brem et al., 2008). It should be mentioned that the wettability of the internal wall and liquid removal from bottles are important factors (Biedermann‐Brem et al., 2008). Indeed, the BPA release from the external face of the baby bottle was higher than the internal face when subjected to detergent which was attributed to different behaviors (Santillana et al., 2013). However, there is some evidence that during the washing or drying cycle, the formed BPA will be removed by water at ambient temperature (Biedermann‐Brem et al., 2008). The point which must be considered is that if rinsing with water fails, the remaining BPA will bake onto the polycarbonate during the drying process (Biedermann‐Brem et al., 2008). Kubwabo et al. (2009) and Mercea (2009) reported that dishwashing had an insignificant effect on BPA migration. The low BPA was attributed to the poor alkalinity of dishwasher solutions and removal of them through rinsing and a short contact period to induce degradation (Kubwabo et al., 2009). Holmes et al. showed that rinsing and hand washing with soap did not influence BPA removal. It was indicated that due to the reduction of BPA during the dishwashing process, the presence of BPA can result from the manufacturing process (Holmes et al., 2021). Despite that, Mercea determined that washing new bottles before using them had insignificant effects on the BPA migration (Mercea, 2009). It could be concluded that BPA produced during the washing or drying cycle is easily removed by rinsing and does not penetrate the polymer (Biedermann‐Brem & Grob, 2009; Maragou et al., 2008), although drying with hard water or maintaining the washing solution combined with high temperature would release BPA on the bottle (Biedermann‐Brem & Grob, 2009). It was also indicated that using ozonized water in the washing process increases the BPA migration (Mercea, 2009).

## 4 CONCLUSION

Many studies calculate the amount of migrated BPA below Tolerable Daily (TDI). However, the fact should be considered that human exposure to BPA is not limited to food containers. There are wide sources of BPA, including the environment (dust particles, fresh water, and air), medicine treatments (dental sealants), cosmetics, toys, paper products (thermal printer paper), digital media products, electronic equipment, and water pipes, which could pose human health into the risk of BPA exposure. Therefore, the use of BPA in baby bottles is banned in some countries, such as Sweden, Brazil, China, Malaysia, France, Denmark, and the USA. Although different studies examined various laboratory techniques to detect the BPA migration, the same results were achieved: repeated use, washing, and rinsing methods, type of detergents, alkaline condition, type of food and packaging, manufacturing process, and irradiation could be effective in BPA leaching. During various conditions, hydrolysis of the carbonate linkage and *d*‐spacing will increase. Also, it is essential to mention that production, transportation, storage, and migration processes are introduced as critical factors in BPA levels. Having investigated different studies, important recommendations for consumers and producers to lower the BPA exposure risk are given as follows:

1. It is recommended to substitute the PC with safer packaging such as PET, HDPE, free‐BPA containers, and lighter forms of glass bottles for baby feeding.
2. If the PC bottles were used to eliminate potential risk, some procedures, such as using sterilization with boiling and steam water, microwaving, and filling bottles with hot water or tea, should be limited. If the boiled water was used to sterilize baby bottles, ensuring that the water is completely poured out and rinsed carefully should be considered.
3. The storage of bottled water under solar irradiation and outdoors should be avoided.
4. During dishwashing with detergents, especially alkaline detergents, rinsing entirely with water and washing correctly should be performed.
5. The manufacturer should state the cleaning and sterilization methods on the label of bottles or packaging. Due to the abundance of plastic container brands, the manufacturer must mention the usage duration on the label to inform the consumer about the appropriate time for discarding the product. Also, the manufacturer should test their products in different conditions to determine the BPA release information and compare them with acceptable TDI.
6. The manufacturer should examine the physicochemical properties of water, including pH, hardness, minerals, cations, and anions, as well as the nature of water before the bottling process. Also, it is essential to monitor the storage condition of bottled or barreled drinking water.
7. Consumers should be aware of discarding the condition of products and label information and replace the aged or old bottles with new ones. The aging process or repeated use of PC bottles leads to surface erosion of bottles and increases the BPA migration.
8. Public awareness about BPA hazards, especially for babies, should be enhanced and teach them to select good brands wisely. It is necessary to consider that if there is no informative label on these products, the bottles should be discarded after changes in color or odor.

## AUTHOR CONTRIBUTIONS

**Elham Khalili Sadrabad:** Conceptualization (equal); data curation (equal); funding acquisition (equal); investigation (equal); methodology (equal); project administration (equal); validation (equal); visualization (equal); writing – original draft (equal); writing – review and editing (equal). **Sayed Aliasghar Hashemi:** Conceptualization (equal); investigation (equal); methodology (equal); resources (equal); writing – original draft (equal); writing – review and editing (equal). **Azadeh Nadjarzadeh:** Data curation (equal); investigation (equal); methodology (equal); project administration (equal); software (equal); supervision (equal); writing – review and editing (equal). **Elaheh Askari:** Conceptualization (equal); investigation (equal); methodology (equal); resources (equal); supervision (equal); writing – review and editing (equal). **Fateme Akrami Mohajeri:** Conceptualization (equal); methodology (equal); project administration (equal); resources (equal); validation (equal); writing – review and editing (equal). **Fereshteh Ramroudi:** Data curation (equal); methodology (equal); resources (equal); visualization (equal); writing – review and editing (equal).

## FUNDING INFORMATION

The authors appreciate the support of the Research Center for Food Hygiene and Safety, Department of Food Hygiene and Safety, School of Public Health, Shahid Sadoughi University of Medical Sciences, Yazd, Iran.

## CONFLICT OF INTEREST STATEMENT

There is no conflict of interest to declare.

## Data Availability

Although the manuscript is a review article, the result of searches is available on request from the corresponding author.
